# Supplementary material for: Maximum Allowed Solvent Accessibilites of Residues in Proteins
Source: PLoS One. 2013 Nov 21;8(11):e80635. doi: 10.1371/journal.pone.0080635 (PMC3836772; doi:10.1371/journal.pone.0080635)
Supplement: File S1 — Combined pdf of Figures S1–S4, Tables S1–S4, and Supporting Text. (PDF) [file pone.0080635.s001.pdf]

Supporting Information for "Maximum allowed solvent accessibilites of residues in proteins"

Matthew Z. Tien, Austin G. Meyer, Dariya K. Sydykova, Stephanie J. Spielman, Claus O. Wilke

Supporting Figures

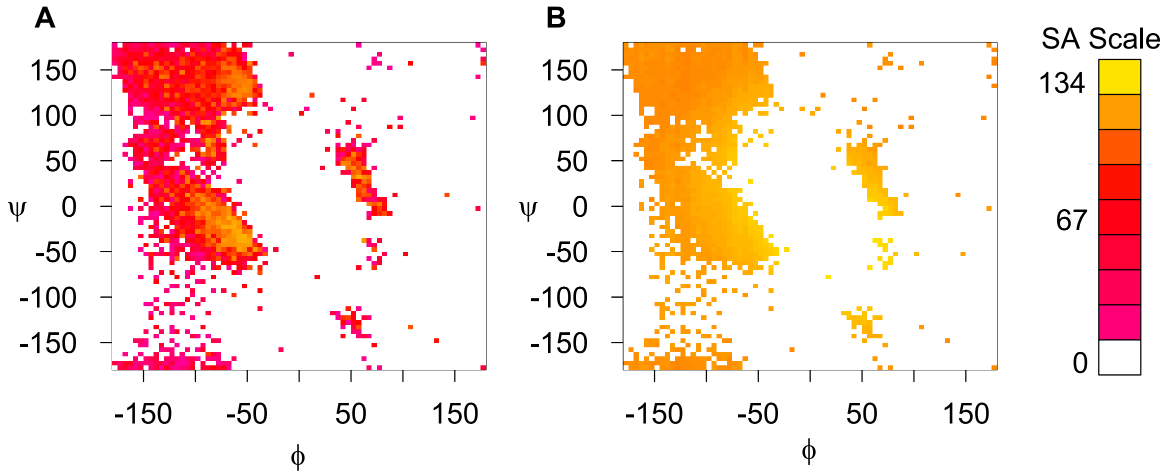

Figure S1. Ramachandran plots for empirical and theoretical maximum SA values of alanine. (A) Empirical maximum SA values for each 5° by 5° bin. All non-empty bins are shown. (B) Theoretical maximum SA values, as determined by computational modeling, shown for non-empty bins in (A).

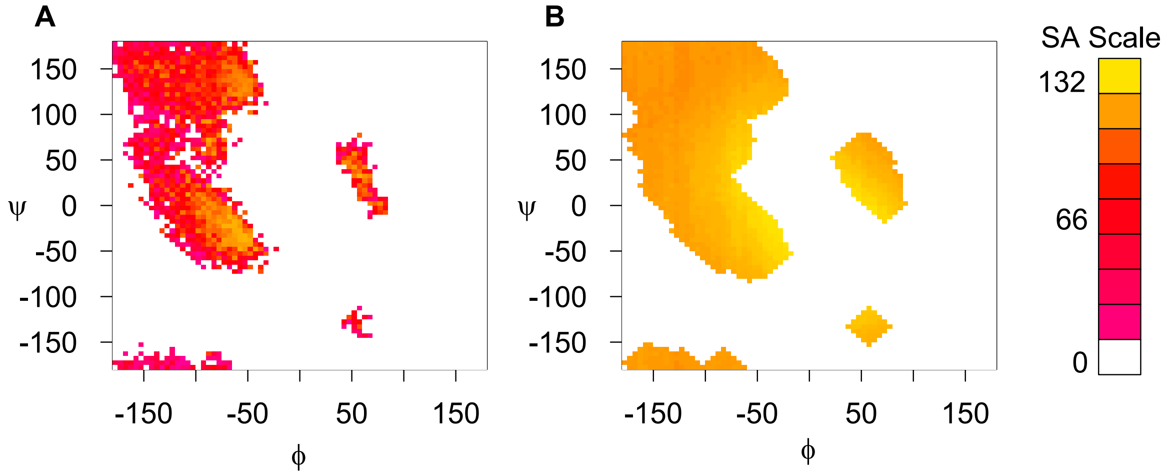

Figure S2. Ramachandran plots for empirical and theoretical maximum SA values of alanine. (A) Empirical maximum SA values for each 5° by 5° bin. All non-empty bins in the GENEROUS regions are shown. (B) Theoretical maximum SA values, as determined by computational modeling, shown for all bins in the GENEROUS region.

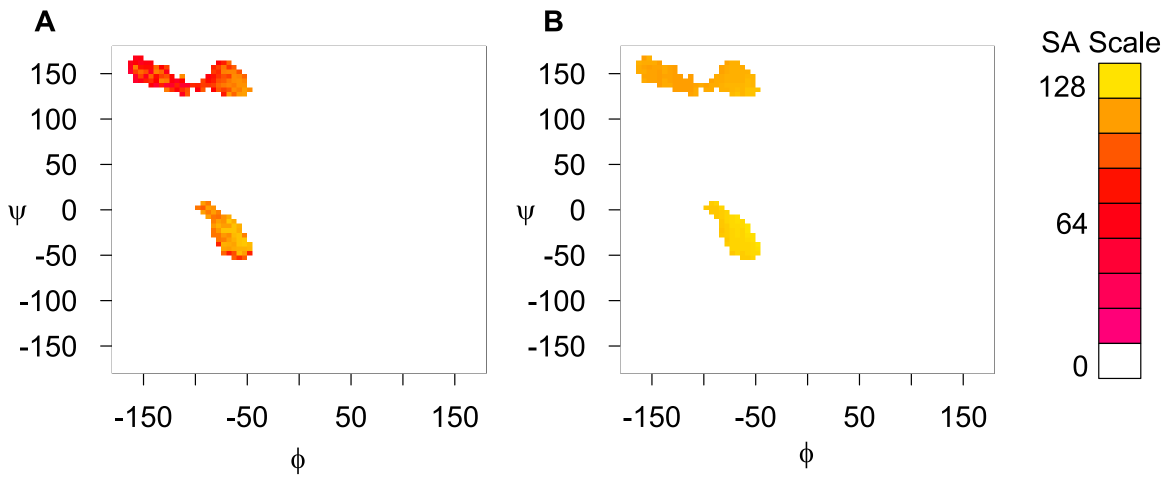

Figure S3. Ramachandran plots for empirical and theoretical maximum SA values of alanine. (A) Empirical maximum SA values for each 5° by 5° bin. All bins in the CORE region are shown. (B) Theoretical maximum SA values, as determined by computational modeling, shown for all bins in the CORE region.

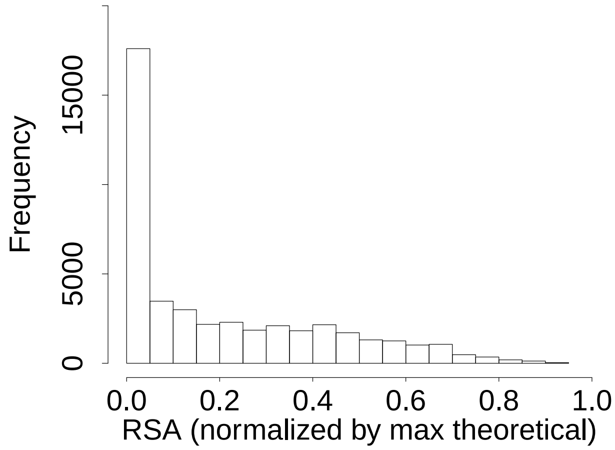

Figure S4. Distribution of RSA values for alanine. RSA was calculated using our theoretically determined normalization values. This distribution is highly non-normal with a strong right skew. Therefore, mean RSA is a poor measure of center for this distribution. Similarly skewed distributions are found for most amino acids.

Supporting Tables

Table S1. Maximum SA values (in Å^2) observed in the empirical and the theoretical data sets.

	Empirical				Theoretical			
Residue	ALL	GENEROUS	ALLOWED	CORE	ALL	GENEROUS	ALLOWED	CORE
Alanine	121	121	121	121	138	132	129	128
Arginine	265	265	265	265	285	280	274	274
Asparagine	187	187	187	187	204	199	195	193
Aspartate	187	187	187	187	204	197	193	192
Cysteine	148	148	148	133	169	167	167	157
Glutamate	214	214	214	214	233	227	223	222
Glutamine	214	214	214	214	234	228	225	224
Glycine	97	97	97	97	114	109	104	104
Histidine	216	216	216	216	231	228	224	219
Isoleucine	195	195	195	195	208	201	197	196
Leucine	191	191	191	191	211	205	201	199
Lysine	230	230	230	229	246	240	236	235
Methionine	203	203	203	203	227	226	224	218
Phenylalanine	228	228	228	226	251	244	240	236
Proline	154	154	154	154	166	164	159	157
Serine	143	143	143	143	161	158	155	150
Threonine	163	163	163	161	182	176	172	171
Tryptophan	264	264	264	262	295	293	285	282
Tyrosine	255	255	255	255	274	266	263	262
Valine	166	166	165	165	184	177	174	173

Table S2. Backbone conformation of maximally exposed trimer structures. Multiple rows per residue indicate alternative conformations with comparable solvent exposure.

	Empirical		Theoretical	
Residue	ϕ	ψ	ϕ	ψ
Alanine	−66.7°	−13.1°	−60°	−15°
	−52.1°	−33.6°		
	−51.9°	−37.9°		
Arginine	−79.2°	−20.2°	−70°	−5°
			−70°	−25°
			−60°	−15° to −10°
			−55°	−30°
			−40°	−50°
Asparagine	−94.8°	−3.4°	−50°	−40°
			−50°	−30°
			70°	−5°
Aspartate	−79.4°	83.0°	70°	−5°
Cysteine	−87.7°	−45.1°	60°	−40°
Glutamate	−55.0°	−49.9°	−60°	−15°
Glutamine	−65.5°	−24.0°	70°	−5°
Glycine	80.2°	7.2°	−75°	20°
			−75°	50°
			−70°	0°
			−65°	−10°
			−60°	−15°
			−50°	−25°
			70°	−15°
			75°	−30°
Histidine	51.2°	32.4°	65°	−25°
			70°	−50°
Isoleucine	−64.1°	−21.9°	−55°	−25°
			−50°	−40°
Leucine	−81.6°	−13.5°	−70°	−5°
	−63.5°	−44.5°		
	−60.9°	−29.3°		
	−55.8°	−36.4°		
Lysine	63.1°	23.1°	−45°	−45° to −40°
Methionine	−67.5°	−27.5°	50°	−40°
Phenylalanine	−50.3°	135.2°	−45°	−40°
			70°	−10°
Proline	−63.8°	−21.6°	−55°	−20°
Serine	−58.1°	−27.3°	65°	−45°
	−103.4°	1.1°		
Threonine	−57.4°	−17.3°	−45°	−45°
Tryptophan	−68.0°	−62.3°	65°	−40° to −35°
			70°	−55°
Tyrosine	−67.6°	−9.8°	−50°	−40° to −35°
			−45°	−45°
Valine	−56.2°	−31.7°	−55°	−25°

Table S3. Hydrophobicity scales derived in this work.

Amino Acid	Mean RSA (theor)[a]	Mean RSA (emp)[b]	100% buried[c]	95% buried[d]
Alanine	0.796	0.782	0.228	0.399
Arginine	0.651	0.639	0.0121	0.0749
Asparagine	0.672	0.658	0.0451	0.146
Asparate	0.646	0.634	0.0276	0.104
Cysteine	0.911	0.899	0.287	0.576
Glutamine	0.654	0.636	0.0289	0.109
Glutamate	0.605	0.589	0.0183	0.0717
Glycine	0.749	0.731	0.166	0.291
Histidine	0.741	0.731	0.0532	0.198
Isoleucine	0.876	0.875	0.247	0.516
Leucine	0.861	0.853	0.213	0.486
Lysine	0.565	0.554	0.00597	0.0283
Methionine	0.856	0.841	0.217	0.484
Phenylalanine	0.87	0.864	0.186	0.483
Proline	0.669	0.658	0.0607	0.162
Serine	0.744	0.722	0.105	0.241
Threonine	0.742	0.728	0.0987	0.237
Tryptophan	0.849	0.837	0.0979	0.368
Tyrosine	0.818	0.813	0.0797	0.306
Valine	0.864	0.857	0.25	0.494

[a]Scale based on mean RSA, as calculated using the theoretially derived SA normalization values for the ALLOWED region. The actual scale is defined as $1 - (\text{mean RSA})$, to yield increasingly larger values for more hydrophobic residues.

[b]Same scale as in (a), but calculated using the empirically derived SA normalization values for the ALLOWED region.

[c]Fraction of 100% buried residues, with RSA = 0 (corresponding to SA < 1Å).

[d]Fraction of 95% buried residues, with RSA < 0.05.

Table S4. Bin cutoffs used to define the ALLOWED (> 97% of data) and the CORE (> 80% of data) regions. For each region and amino acid, bins with as many or fewer observations as listed were discarded.

Residue	ALLOWED	CORE
Alanine	4	47
Arginine	2	23
Asparagine	2	15
Aspartate	3	20
Cysteine	0	5
Glutamate	3	34
Glutamine	2	18
Glycine	2	15
Histidine	1	9
Isoleucine	6	40
Leucine	7	59
Lysine	3	27
Methionine	0	8
Phenylalanine	2	20
Proline	7	80
Serine	2	27
Threonine	3	33
Tryptophan	0	8
Tyrosine	2	18
Valine	7	50

Supporting Text – Tripeptide construction

We construct tripeptides by placing atoms one-by-one at the correct location in 3D space. We always begin with the N-terminus residue, which we place at the origin: The α carbon is placed at coordinates $(0, 0, 0)$, and the carbonyl carbon is placed at $(1.52, 0, 0)$, reflecting the 1.52Å bond length between a carbonyl group and a carbon atom. Next, the nitrogen atom is placed at $(\ell \cos\theta, \ell \sin\theta, 0)$, where ℓ is the bond length and θ is the bond angle. To ensure that the constructed residue is in the L-conformation, the nitrogen atom is rotated positively from the x-axis.

All subsequent atoms are placed using the following procedure: We identify three reference atoms, which we refer to as A, B, and C. We translate these points such that C lies at the origin. We then calculate the coordinates (x, y, z) of a fourth atom X at a bond length ℓ from C, a bond-angle θ relative to the CB vector (which we refer to as $\mathbf{b}$), and a dihedral angle τ relative to the ABC plane (see figure on next page). In this calculation, we proceed in two steps: We initially place the atom at the correct distance from C and correct bond angle relative to the CB vector, but with an arbitrary dihedral angle. We then rotate the atom around the CB vector to place it at the correct dihedral angle.

To achieve the initial placement, we solve a system of three equations, corresponding to the three conditions that the distance from C to X should be ℓ, the angle between CB and CX be θ, and that X be in a plane perpendicular to ABC:

$$x^2 + y^2 + z^2 = \ell^2, \tag{1}$$

$$\mathbf{b} \cdot (x, y, z) = \ell ||\mathbf{b}|| \cos\theta, \tag{2}$$

$$Ix + Jy + Kz = 0. \tag{3}$$

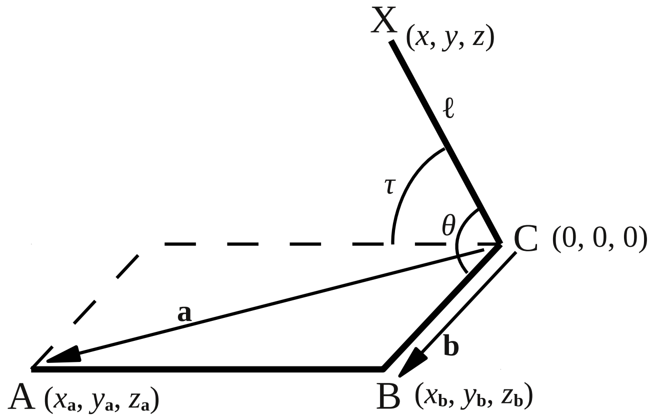

Schematic drawing of the placement of an atom relative to three reference atoms A, B, and C. Atom C is assumed to be at the origin. The vector from C to B is denoted by $\mathbf{b}$, with coordinates $(x_\mathbf{b}, y_\mathbf{b}, z_\mathbf{b})$. Similarly, the vector from C to A is denoted by $\mathbf{a}$, with coordinates $(x_\mathbf{a}, y_\mathbf{a}, z_\mathbf{a})$. The distance from C to X is ℓ, the angle between CB and CX is θ, and the dihedral angle between ABC and CX is τ.

Here, the constants I, J, and K are coefficients of the target plane, and are obtained from the cross product of vectors **a** and **b**:

$$
\begin{aligned}
I &= (y_{\mathbf{a}} z_{\mathbf{b}}) - (z_{\mathbf{a}} y_{\mathbf{b}}), & (4)\\
J &= (z_{\mathbf{a}} x_{\mathbf{b}}) - (x_{\mathbf{a}} z_{\mathbf{b}}), & (5)\\
K &= (x_{\mathbf{a}} y_{\mathbf{b}}) - (y_{\mathbf{a}} x_{\mathbf{b}}). & (6)
\end{aligned}
$$

Solving for x, y, and z yields the following expressions:

$$x = \frac{R - IJPy_{\mathbf{b}} + PK(Kx_{\mathbf{b}} - Iz_{\mathbf{b}}) + J^2Px_{\mathbf{b}}}{Q}, \tag{7}$$

$$y = \frac{-I(Rz_{\mathbf{b}} + J^2Px_{\mathbf{b}}z_{\mathbf{b}} - JPKx_{\mathbf{b}}y_{\mathbf{b}}) + K[Rx_{\mathbf{b}} - P(Jz_{\mathbf{b}} - Ky_{\mathbf{b}})^2] + I^2Py_{\mathbf{b}}(Jz_{\mathbf{b}} - Ky_{\mathbf{b}})}{Q(Jz_{\mathbf{b}} - Ky_{\mathbf{b}})}, \tag{8}$$

$$z = \frac{IRy_{\mathbf{b}} - JRx_{\mathbf{b}} + I^2Pz_{\mathbf{b}}(Jz_{\mathbf{b}} - Ky_{\mathbf{b}}) + IPKx_{\mathbf{b}}(Ky_{\mathbf{b}} - Jz_{\mathbf{b}}) + JP(Jz_{\mathbf{b}} - Ky_{\mathbf{b}})^2}{Q(Jz_{\mathbf{b}} - Ky_{\mathbf{b}})}, \tag{9}$$

where constants Q and R are defined as

$$Q = I^2(y_{\mathbf{b}}^2 + z_{\mathbf{b}}^2) + J^2(x_{\mathbf{b}}^2 + z_{\mathbf{b}}^2) + K^2(x_{\mathbf{b}}^2 + y_{\mathbf{b}}^2) - 2Jy_{\mathbf{b}}(Ix_{\mathbf{b}} + Kz_{\mathbf{b}}) - 2IKx_{\mathbf{b}}z_{\mathbf{b}}, \tag{10}$$

$$R = \sqrt{(Jz_{\mathbf{b}} - Ky_{\mathbf{b}})^2[Q\ell^2 - P^2(I^2 + J^2 + K^2)]}. \tag{11}$$

As we can see from the denominator in Equations (8) and (9), the expressions for y and z are undefined if both $y_{\mathbf{b}}$ and $z_{\mathbf{b}}$ are zero. In this case, the appropriate solution is

$$y = \frac{-IJx + S}{J^2 + K^2}, \tag{12}$$

$$z = \frac{-IK^2x + JS}{K(J^2 + K^2)}, \tag{13}$$

with x given by equation (7) and S defined as

$$S = \sqrt{K^2[-I^2x^2 + J^2K^2(\ell - x)(\ell + x)]}. \tag{14}$$

These equations for x, y, and z yield two possible solutions; the first corresponds to a dihedral angle of 0° from ABC, and the second corresponds to a dihedral angle of 180° from ABC. We arbitrarily selected one of these solutions. We then rotated the point X around **b** until it was located at the appropriate dihedral angle τ relative to ABC.

Once atom X is placed at position (x, y, z), we translate these coordinates back to the original coordinate system in which C is not at the origin.
